# Supplementary material for: Cell-free reconstitution reveals centriole cartwheel assembly mechanisms
Source: Nat Commun. 2017 Mar 23;8:14813. doi: 10.1038/ncomms14813 (PMC5376648; doi:10.1038/ncomms14813)
Supplement: Supplementary Information — Supplementary Figures [file ncomms14813-s1.pdf]

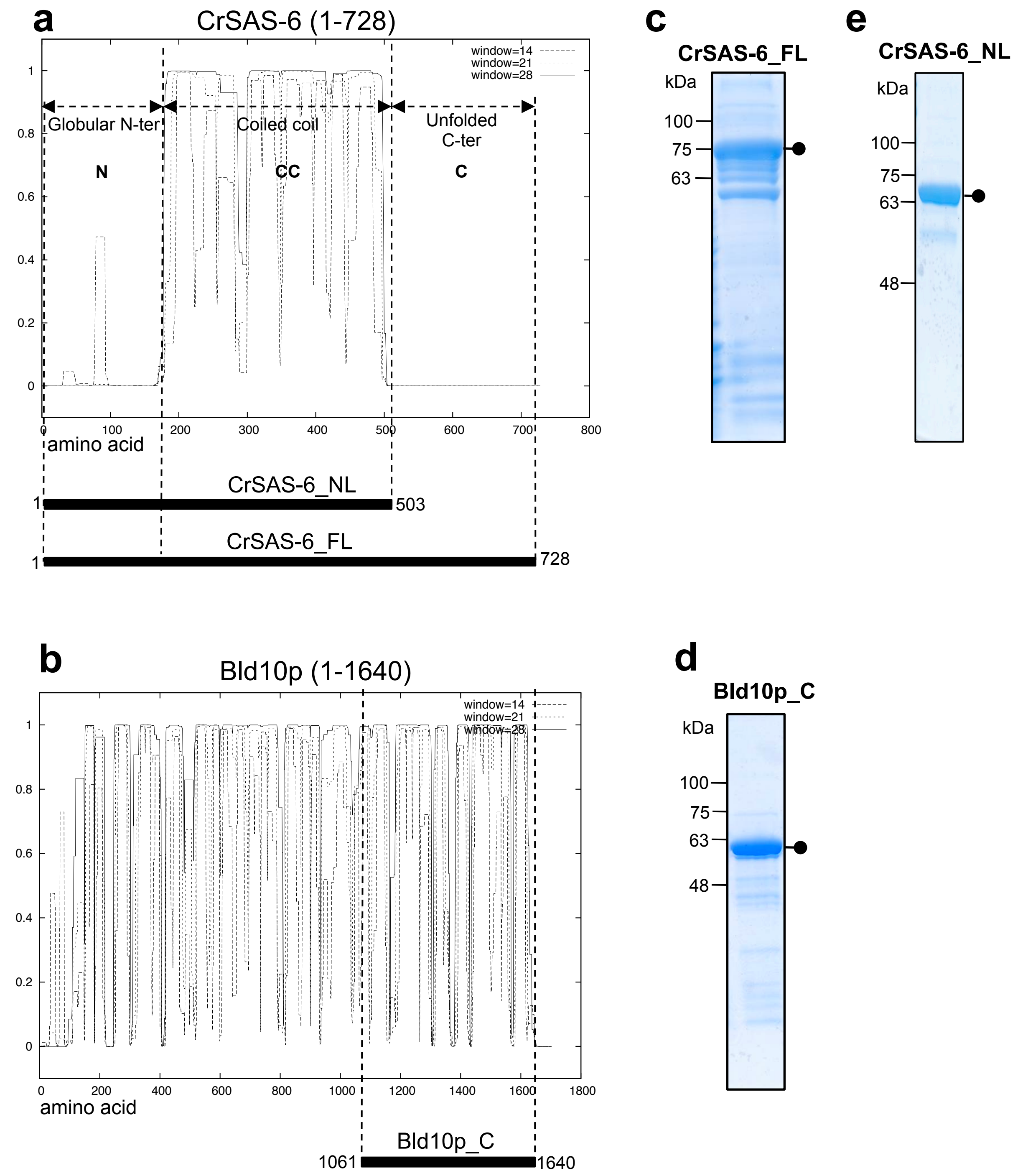

Supplementary Figure 1: CrSAS-6 and Bld10p proteins.

a-b, Coiled coil prediction of full-length CrSAS-6 (a) and full-length Bld10p (b) using the COILS software, with corresponding schematic representations. Note that the distance encompassing the coiled coil of CrSAS-6_CC is predicted to be ~45 nm (~300 residues × 0.1485 nm [axial raise per residue] = ~44.55 nm). **c-e**, SDS-PAGE of recombinant proteins used in this study: HisCrSAS-6_FL (c), Bld10p_C (d**)** and His-StagCrSAS-6_NL (e). Molecular size markers are indicated on the left in kDa; proteins of interest are marked by a dot.

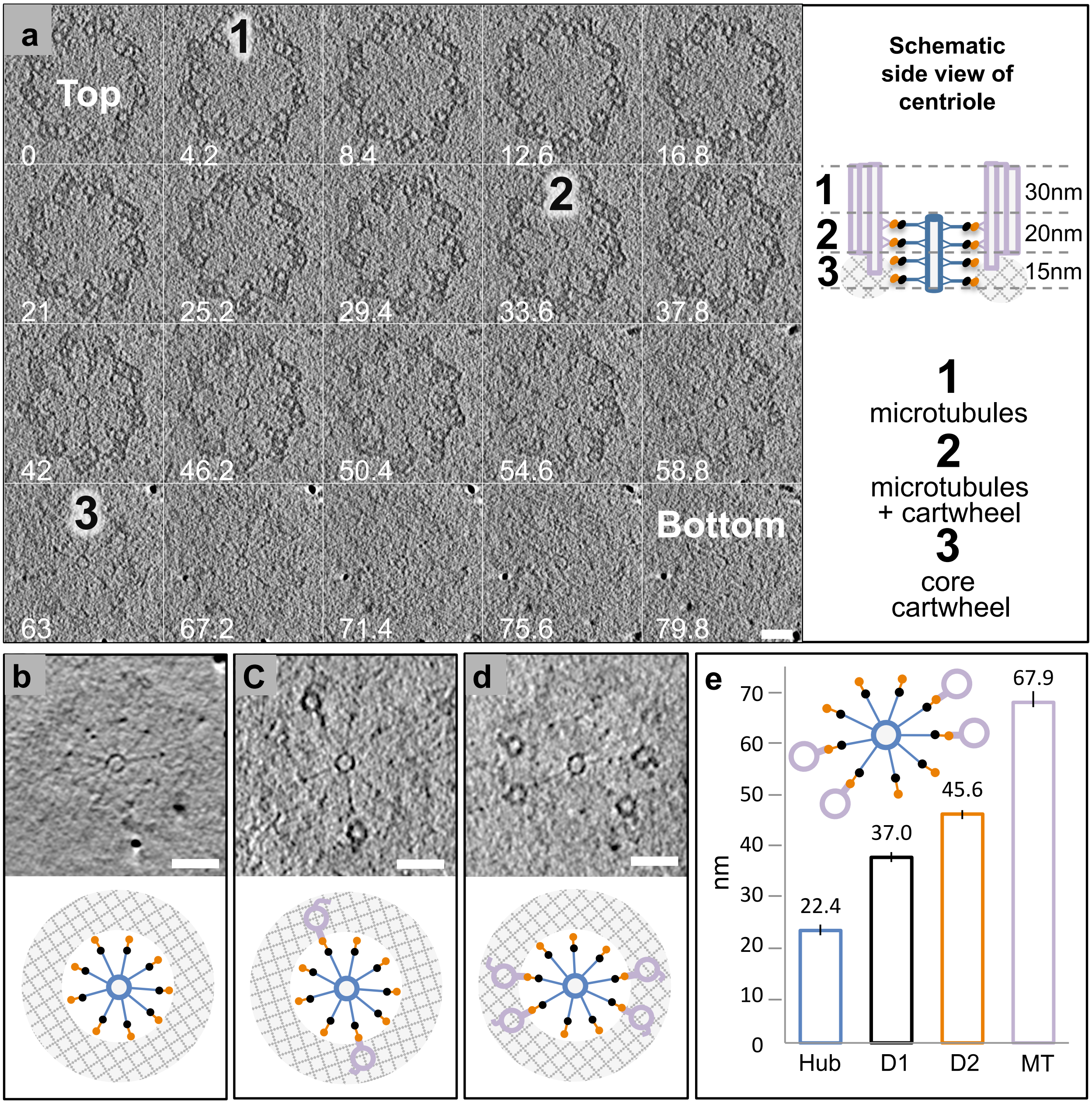

Supplementary Figure 2: Cryo-electron tomogram of *Chlamydomonas* procentriole.

a, Raw images extracted from a cryo-electron tomogram of ~65 nm-proximal most part of a *Chlamydomonas* centriole (probasal body, schematized representation on the right), revealing three regions with distinct features, from top to bottom: (1) microtubules only, without cartwheel (top 30 nm); (2) microtubules, pinhead and associated cartwheel (next 20 nm); (3) core cartwheel (bottom 15 nm). The white numbers indicate the distances in nm in the z axis. **b**, High magnification view of core cartwheel surrounded by an amorphous ring (top) and corresponding schematic representation (bottom). **c, d**, Procentrioles harboring a core cartwheel with none (b), two (c) or four (d) A-microtubules attached to distinct spokes (top) and corresponding schematic representations (bottom). **e**, Measurements of centriole features: hub diameter (22.4 +/- 1.3 nm, n= 10), distances from the hub margin to D1 (37 +/- 1 nm, n= 13) and D2 (45.6 +/- 0.8 nm, n= 13), as well as distance from the hub margin to the edge of the A-microtubule (67.9 +/- 2.3 nm, n= 13). Data from 5 tomograms. All scale bars: 50 nm.

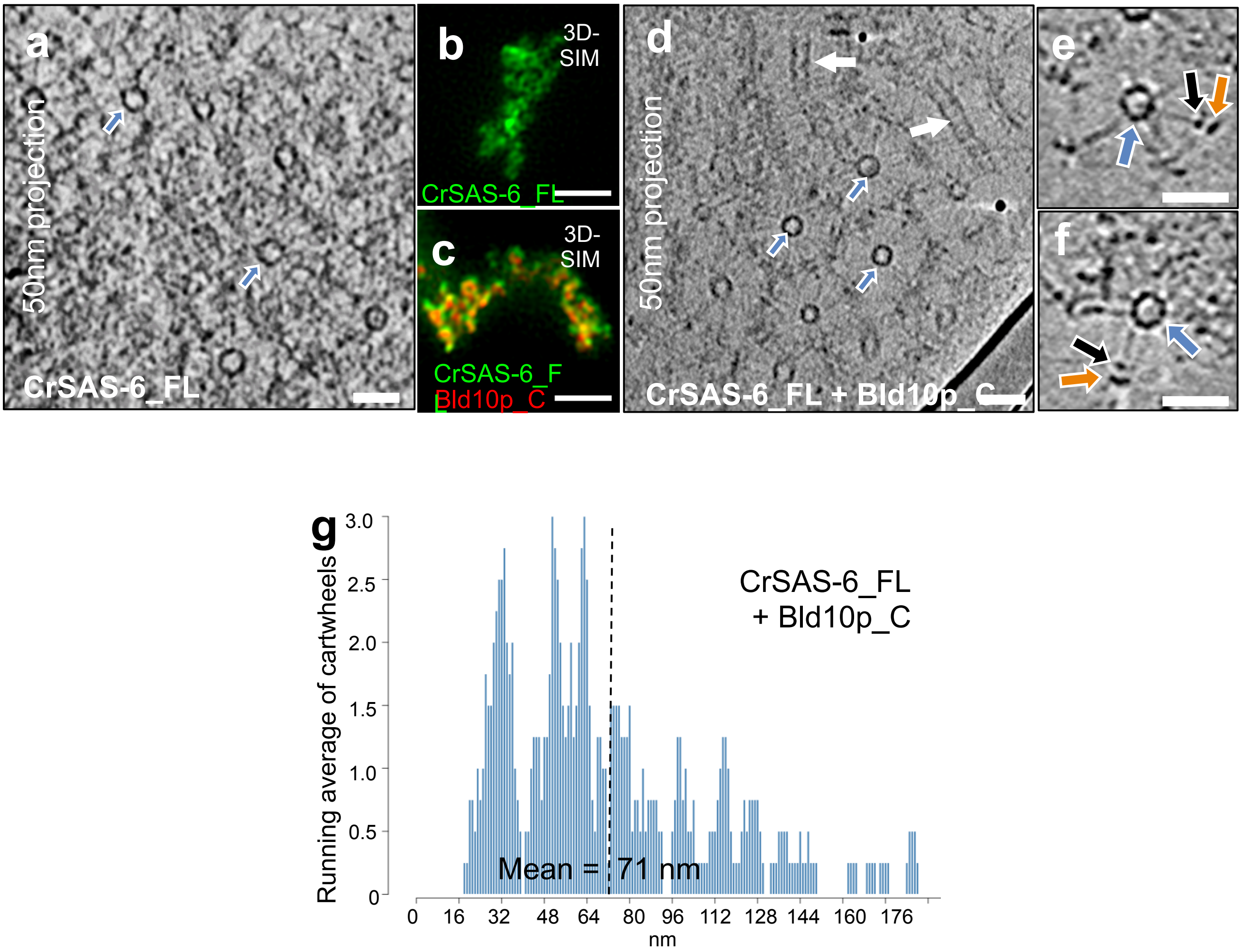

Supplementary Figure 3: CrSAS-6_FL and Bld10p_C interact and form 3D core cartwheels *in vitro.*

a, Cryo-electron tomogram of assemblies formed by CrSAS-6_FL. Blue arrows indicate SAS-6 rings. Scale bars: 50 nm.**b, c,** Structured illumination microscopy **(**SIM) images of indicated proteins spun onto coverslips to reveal assemblies formed by CrSAS-6_FL alone (b) or by CrSAS-6_FL/Bld10p_C (c); coverslips were stained with the indicated antibodies (against CrSAS-6, green, (b) and (c), as well as against Bld10p, red, (c) only). Scale bar: 2 µm. **d**, Cryo-electron tomogram of assemblies formed by CrSAS-6_FL/Bld10p_C. Note side views of stacked cartwheel rings, appearing as tubes (arrows). **e, f,** High magnification view of two core cartwheels showing the hub (blue arrows), D1 (black arrows) and D2 (orange arrows). Note that the spokes are not always arranged as in the native cartwheel and can be parallel to one another, presumably due to spatial constraints in the lattice. **g**, Height distribution of cartwheels reconstituted with CrSAS-6_FL/Bld10p_C. Peak values are indicated on the graph and the dotted line corresponds to the mean (average: 71 +/- 38 nm, n= 130 cartwheels, data from 2 tomograms). Scale bars: 50 nm.

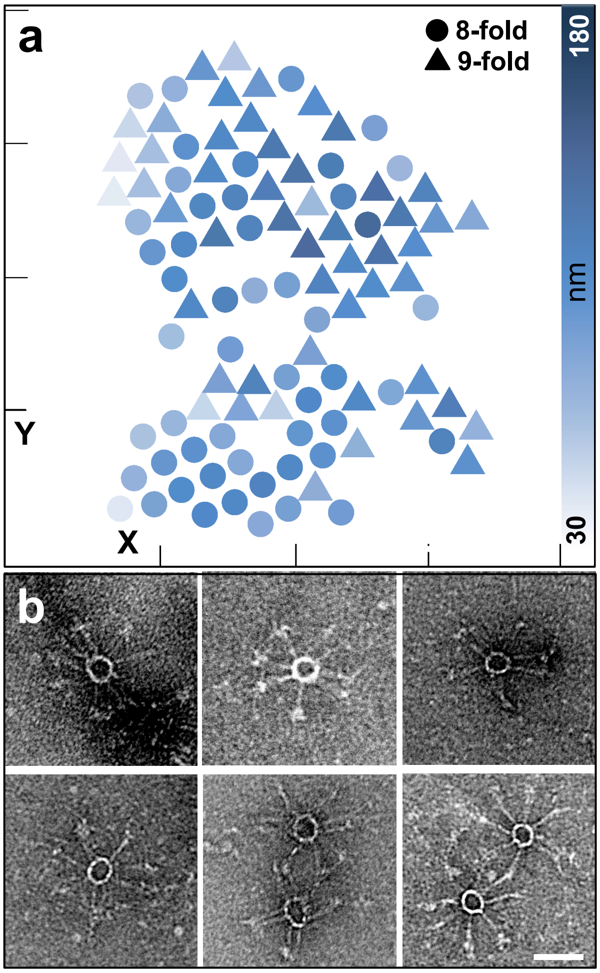

Supplementary Figure 4: Cartwheel height distribution in the lattice and correspondence of D1 with the C-terminal end of CrSAS-6_NL

a, Heat map representation of cartwheel heights with XY coordinates in one tomogram, along with indication of 8-fold and 9-fold symmetries. **b**, Further examples of CrsAS-6_NL assemblies viewed using negative stain EM, showing presence of hub, spokes and D1 density. Scale bars: 50 nm.

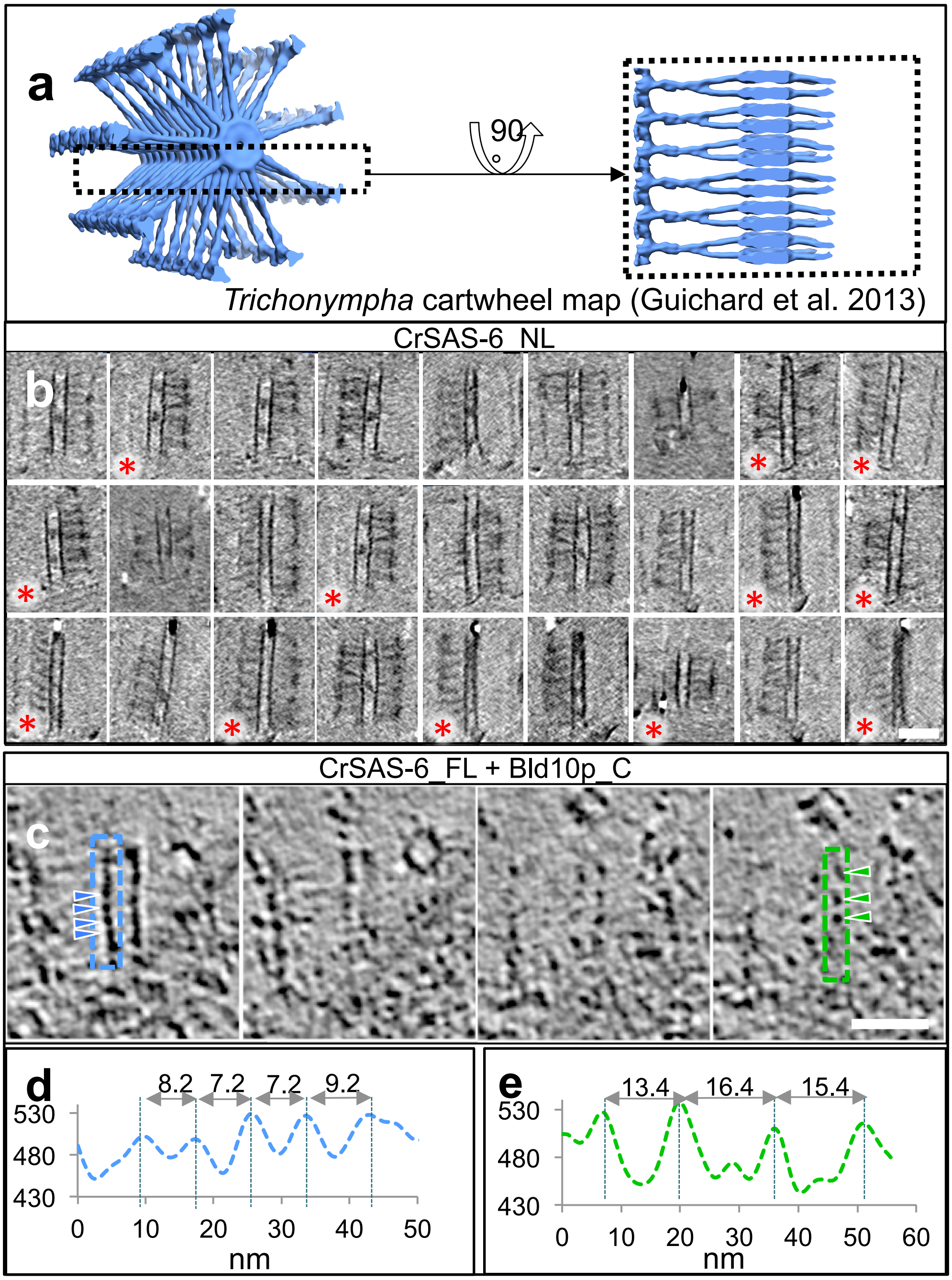

Supplementary Figure 5: Periodicities along the periphery of radial spokes in reconstituted CrSAS-6_NL and CrSAS-6_FL/Bld10p_C cartwheels.

a, 3D representation of the *Trichonympha sp*. cartwheel EM map, showing spokes emanating from the hub and merging towards the periphery[9]. **b**, Z-axis images extracted from one tomogram of CrSAS-6_NL assemblies, exemplifying the periodicity visible along the periphery of the spokes (n=28 cartwheels). Note that the low resolution in the Z-axis is due to the missing wedge inherent to electron tomographic reconstruction. Red asterisks indicate images used to extract peripheral spokes periodicities. **c**, Images extracted from a cryo-electron tomogram with CrSAS-6_FL/Bld10p_C, showing spoke periodicities at the hub margin (blue) and at the periphery (green). **d, e,** Measurements of periodicities at the hub margin ((d), average: 8.4 nm +/- 1.6, N=24 measurements from 5 cartwheels) and at the periphery ((e), average: 15.1 +/- 1.5 nm, N=3 measurements from 1 cartwheel). Note that CrSAS-6_FL/Bld10p_C cartwheel spokes are not organized perfectly laterally, thus limiting the number of possible measurements. Scale bar: 50 nm.

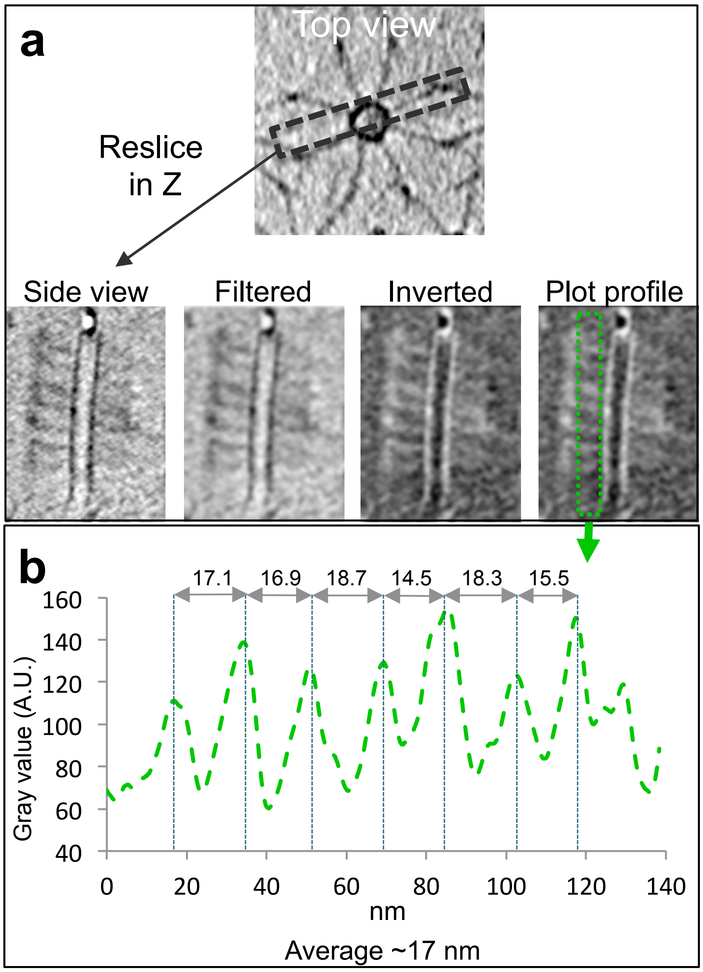

Supplementary Figure 6: Example of periodicity measurement along the radial spokes.

a, CrSAS-6_NL cartwheel extracted from the tomogram resliced in Z in order to observe the periodicity along the radial spokes, processed with a Gaussian filter, and inverted to facilitate data analysis. The plot profile has been extracted from the region boxed in green. **b,** Measurements of periodicities between spokes using ImageJ plot. The average value is 17nm +/- 1.6 (n=6, data from 1 tomogram).

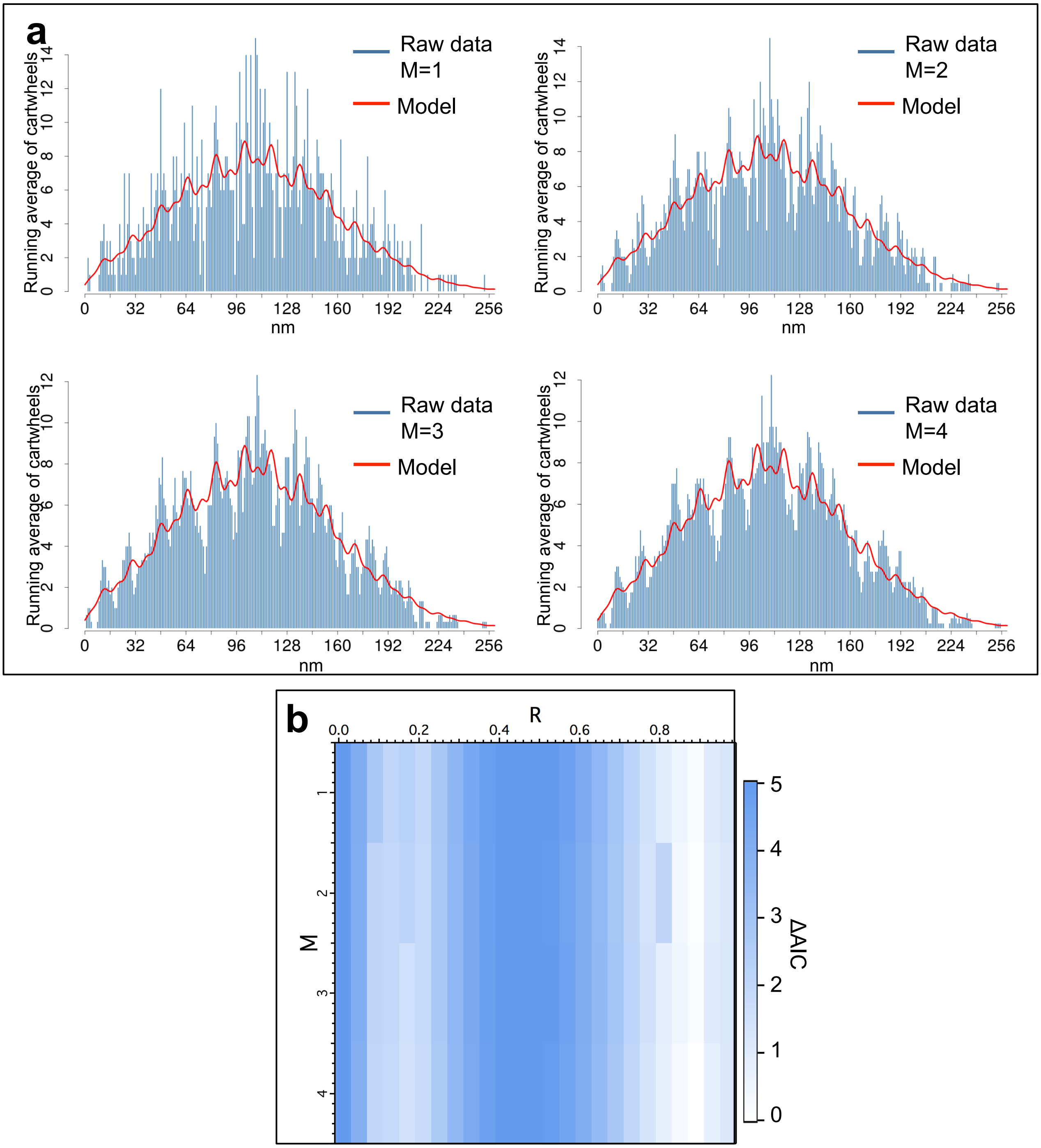

Supplementary Figure 7: Fit of the model using different running average parameters.

a, Height distribution of CrSAS-6_NL reconstituted cartwheels smoothed with no running average (M=1), average of 2 (M=2), 3 (M=3) and 4 (M=4). The red line corresponds to the fit of the model on the experimental data. **b**, The AIC differences (ΔAIC) corresponding to the (h_1,h_2) pairs minimizing the AIC computed at varying offsets (s) and abundance ratios (R) as a function of different running averages M.

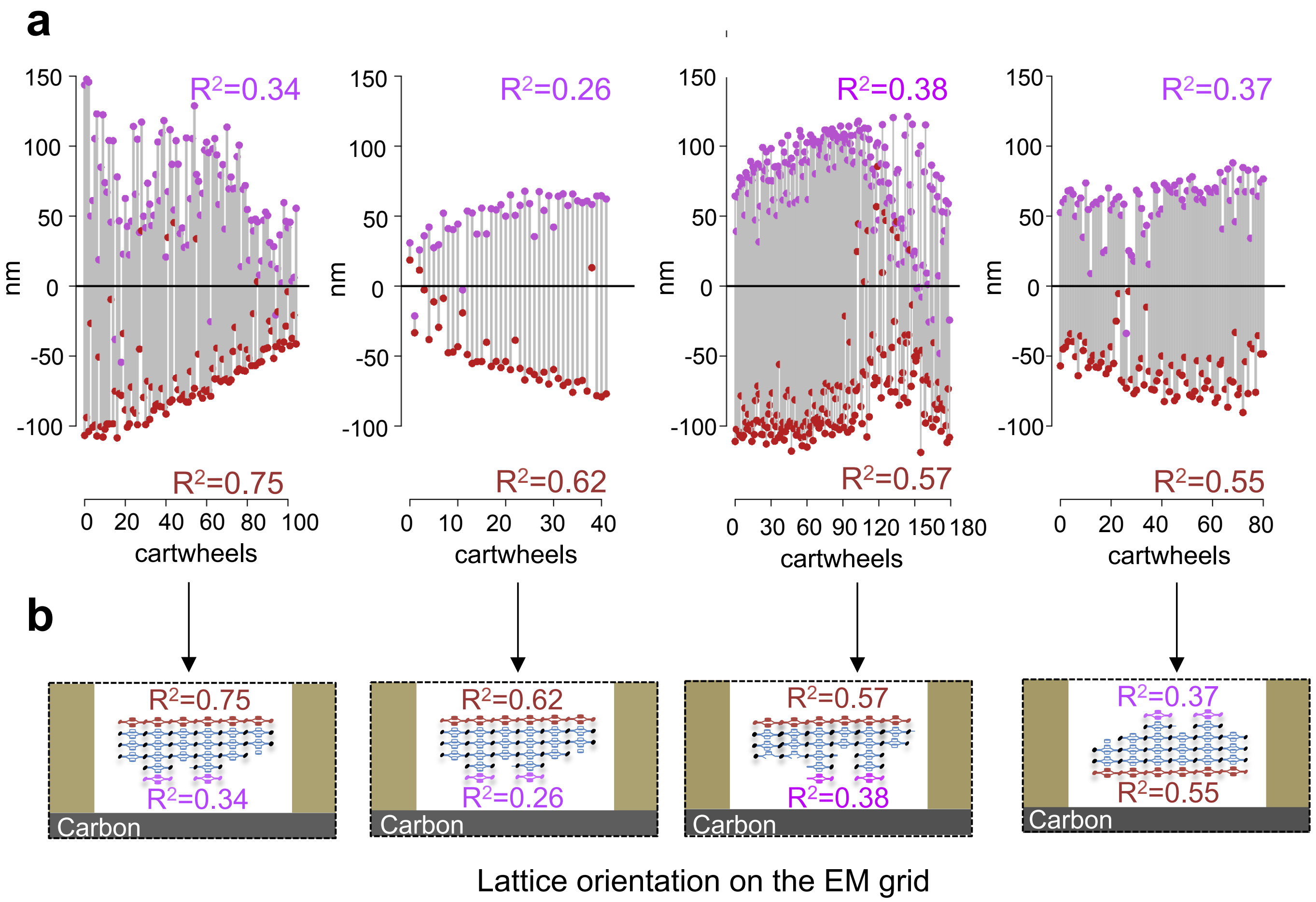

Supplementary Figure 8: Directional growth of CrSAS-6_NL cartwheels within the crystal

a, Distances from a global regression plane (central line) of the two extremities of individual cartwheels within four tomograms (tomogram 1, n= 105 cartwheels, tomogram 2, n=42 cartwheels, tomogram 3, n=180 cartwheels, tomogram 4, n=81 cartwheels). Regression planes for the two extremities were computed and corresponding R^2 values calculated (lower R^2 values=0.34, 0.26, 0.38 and 0.37 respectively, violet; corresponding higher R^2 values=0.75, 0.62, 0.57 and 0.55 respectively, red). **b**, Schematic representation of lattice orientation with respect to the surface of the EM grid. Note that three lattices (left-most three) are oriented with the more heterogeneous side towards the holey carbon support film of the EM grid and one (right-most) with the more homogenous side towards the holey carbon support film of the EM grid.
